# Pancreato-jejunostomy versus hand-sewn closure of the pancreatic stump to prevent pancreatic fistula after distal pancreatectomy: a retrospective analysis

**DOI:** 10.1186/1471-2482-13-23

**Published:** 2013-07-02

**Authors:** Roberto L Meniconi, Roberto Caronna, Dario Borreca, Monica Schiratti, Piero Chirletti

**Affiliations:** 1 Department of Surgical Sciences, Sapienza University of Rome, Viale del Policlinico 155, Rome 00161, Italy

**Keywords:** Distal Pancreatectomy, Pancreatic Fistula, Pancreato-jejunostomy, Roux-en-Y, Hand-sewn Closure

## Abstract

**Background:**

Different methods of pancreatic stump closure after distal pancreatectomy (DP) have been described to decrease the incidence of pancreatic fistula (PF) which still represents one of the most common complications in pancreatic surgery. We retrospectively compared the pancreato-jejunostomy technique with the hand-sewn closure of the pancreatic stump after DP, and analyzed clinical outcomes between the two groups, focusing on PF rate.

**Methods:**

Thirty-six patients undergoing open DP at our institution between May 2005 and December 2011 were included. They were divided in two groups depending on pancreatic remnant management: in 24 cases the stump was closed by hand-sewn suture (Group A), while in 12 earlier cases a pancreato-jejunostomy was performed (Group B). We analyzed postoperative data in terms of mortality, morbidity and length of hospital stay between the two groups.

**Results:**

PF occurred in 7 of 24 (29.1%) cases of group A (control group) compared to zero fistula rate in group B (anastomosis group) (p=0.005). Operative time was significantly higher in the anastomosis group (p=0.024). Mortality rate was 0% in both groups. Other postoperative outcomes such as hemorrhages, infections, medical complications and length of hospital stay were not significant between the two groups.

**Conclusion:**

Despite a higher operative time, the pancreato-jejunostomy after DP seems to be related to a lower incidence of PF compared to the hand-sewn closure of the pancreatic remnant.

## Background

Distal pancreatectomy (DP) is a surgical procedure performed mostly for benign, borderline or malignant tumors of the body and tail of the pancreas [1]. It is also indicated for the treatment of chronic pancreatitis [2]. Depending on the disease, it could be associated to splenectomy, lymphadenectomy or multivisceral resections. Despite this operation is performed with relatively low morbidity and mortality rates in high-volume centers, the leakage from pancreatic stump after DP remains a problem, determining a pancreatic fistula (PF) in 5-30% of cases according to recent papers [1,3,4] and contributing to increased morbidity and overall costs. Different techniques of pancreatic stump closure have been described to reduce the incidence of PF, such as stapler transection, pancreatic duct occlusion by fibrin-glue sealant, serosal or artificial patches, ultrasonic scalpel or radiofrequency dissector [1,5-10], but none has proved to be the most effective in preventing PF. Up till now, few authors described the drainage of the pancreatic stump into a jejunal loop [5,11-13] and a recent study demonstrated a significant decrease of pancreatic leakage by performing a Roux-en-Y pancreato-jejunostomy [14]. The aim of this study is to confirm the efficacy of the pancreato-jejunostomy in reducing pancreatic fistula rate after DP, compared to simple hand-sewn closure of the pancreatic remnant.

## Methods

A total of 36 patients (14 males and 22 females) undergoing DP between May 2005 and December 2011 were included in this study and retrospectively analyzed. All patients were studied preoperatively by contrast-enhanced computed tomography or magnetic resonance imaging. Indications for surgery were benign, borderline or malignant tumors, chronic pancreatitis and pancreatic pseudocysts. Surgical operation consisted in an en-bloc resection of the pancreas tail, eventually extended to the body, associated with splenectomy or other organs resection if needed. In all cases an open approach was performed by a single surgeon. Most of pancreato-jejunostomies were performed not consecutively in the first period of this study, between May 2005 and October 2008, depending on the surgeon preference. From November 2008 all patients undergoing DP were enrolled in another survey in which the pancreatic stump was closed by direct suture with the technique described below. Then we retrospectively observed and analyzed different outcomes between the two techniques.

**Figure 1:**
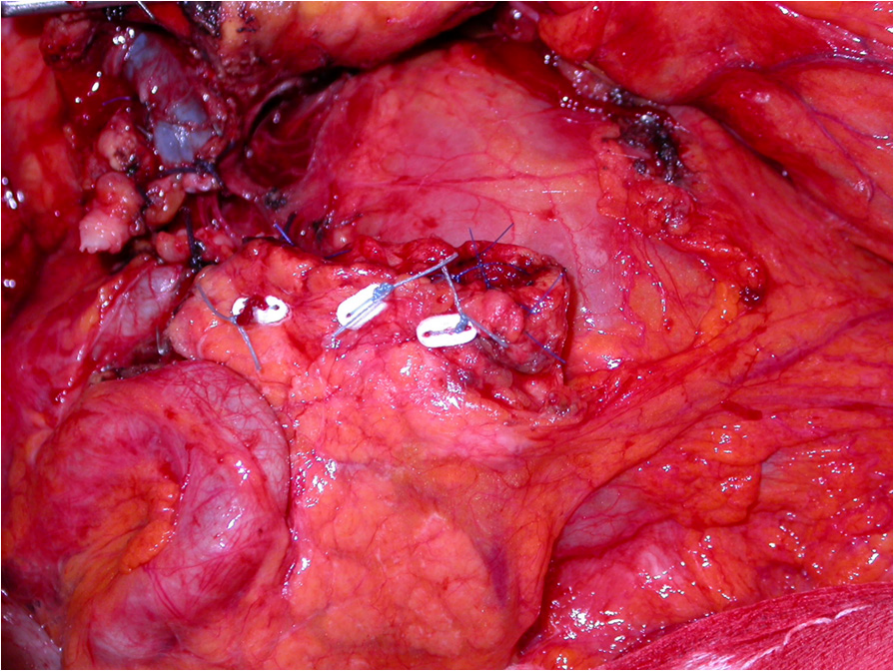
**Hand-sewn closure of the pancreatic remnant.** The pancreatic remnant is closed using PTFE pledget-supported interrupted stitches of non-resorbable material.

**Figure 2:**
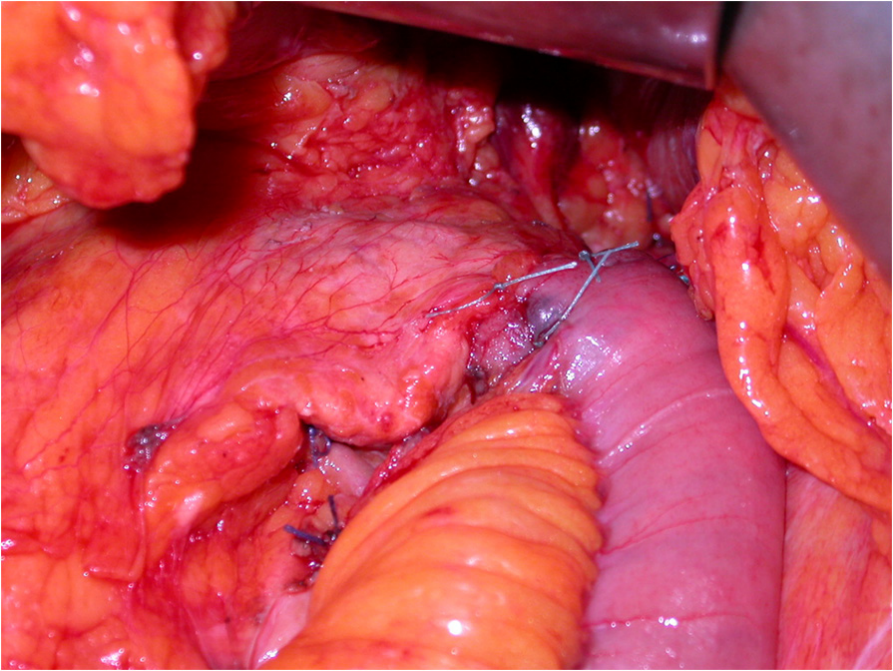
**Roux-en-Y end-to-end pancreato-jejunostomy.** The pancreatic stump is invaginated into the jejunal loop and a capsule-to-seromuscular suture is performed using non-resorbable interrupted stitches.

Patients were divided in two groups on the basis of pancreatic stump management. In the first group (Group A), after pancreatic resection, the stump closure was accomplished by ligating the main pancreatic duct with non-resorbable Z-shaped suture and the cut margin was over sewn a traumatically by U-shaped stitches using non-resorbable material (TiCron®, Covidien, Mansfield, MA, USA) supported by PTFE (Teflon) pledgets used as buttress for the suture (Figure 1). In the second group (Group B), the main pancreatic duct was closed with the same technique described above and the pancreatic stump was finally invaginated into a jejunal loop performing a Roux-en-Y end-to-end pancreato-jejunostomy. The anastomosis was completed by a capsule-to-seromuscular single layer suture with non-resorbable interrupted stitches (Figure 2). A drain was placed intraoperatively in all cases near the anastomosis or the pancreatic stump. All patients received a short-term antibiotic prophylaxis.

Intravenous fluids, octreotide (3 × 0.1 mg s.c., daily for 5–7 days) and proton pump inhibitors (omeprazole, 40 mg i.v., daily) were administrated postoperatively. Oral feeding was generally resumed depending on gastrointestinal function. Drainage volume and amylase concentration of drained fluid were measured and registered in the 1^st^, 3^rd^, 5^th^ and 7^th^ postoperative day as well as blood tests.

Patient demographics, operation data, post-operative morbidity, mortality rate and length of hospital stay were analyzed and compared between the two groups. PF was defined as a drain output of any measurable volume of fluid on or after postoperative day 3 with an amylase content greater than 3 times the upper normal serum value, in accordance to the International Study Group of Pancreatic Fistula (ISGPF) [15]. PF was also classified into three grades (A,B,C) depending on clinical impact (none, moderate and severe, respectively), according to the ISGPF classification. The pancreatic texture was defined as “fibrotic” or “non-fibrotic” depending on histological findings of the specimens: presence of perilobular fibrosis, chronic inflammatory reactions with ductal dilatations, atrophy of the acinar cells. The main pancreatic duct (MPD) was also defined as “small” or “large” according to the diameter < or > 3 mm, respectively. Postoperative hemorrhage (PH) was defined and classified into three different grades (A,B,C), according to the International Study Group of Pancreatic Surgery definition (ISGPS) [16]. Mortality was considered as any death occurred intraoperatively or during the hospital stay.

Statistical analysis was performed using χ^2^ test and Student’s t-test. Differences were considered significant at p-value <0.05.

The present study is a retrospective review of medical data and the human research ethics committee of our institution stated to exempt it from formal ethical review according to the ethical principles laid forth by the Helsinki Declaration. Written consent of patients was not sought. No identifying information was recorded by the authors.

## Results

Between May 2005 and December 2011, thirty-six patients underwent DP at our institution. All patients were divided retrospectively in two groups depending on pancreatic stump management: Group A (control group) comprised 24 patients with a mean age of 53.6 years (range 25–75 years) in which the pancreatic remnant was closed by an hand-sewn technique; Group B (anastomosis group) comprised 12 patients with a mean age of 50.5 years (range 27–67 years) in which a pancreato-jejunostomy was performed. Patients demographics of two groups were compared and well-matched as reported in Table 1. Indications for surgical resection were pancreatic tumors in 30 patients (20 of group A, 10 of group B), chronic pancreatitis in 3 patients (2 of group A and 1 of group B), and pancreatic pseudocysts in 3 patients (2 of group A, 1 of group B). Splenectomy was performed in 22 cases (61,1%), while a cholecystectomy was carried out in 4 patients with gallstones; two cases required a left nephrectomy due to a locally advanced disease and in one case a portal vein resection with graft reconstruction was performed for suspicion of neoplastic venous infiltration. Mean operative time was significantly higher in the anastomosis group (192 min) compared to the control group (161 min) (p=0.024). 10 patients (83,6%) of the anastomosis group had a non-fibrotic pancreas compared to 18 patients (75%) of the control group (p=0.584); a small MPD was found in 15 patients of group A and in 8 patents of group B (p=0.622). All operative data are summarized in Table 2.

**Table 1:** Patient demographics

**Data**	**Group A**	**Group B**	**P-value**
	**(control; *n *=24)**	**(anastomosis; *n *=12)**	
Age (years)	53.6 (25–72)	50.5 (27–67)	0.473
Gender			0.640
- Male	10	4	
- Female	14	8	
BMI	29.6 (24.1-40.5)	28.9 (22.5-35.4)	0.681
Tobacco use	11	5	0.819
Alcohol abuse	6	4	0.611
DM	8	5	0.635
HTN	11	5	0.819
COPD	1	1	0.619
CRF	1	0	0.487

All quantitative values are given as mean (range).

*BMI* Body Mass Index, *DM* diabetes mellitus, *HTN* arterial hypertension, *COPD* chronic obstructive pulmonary disease, *CRF* chronic renal failure.

**Table 2:** Operation data

**Data**	**Group A**	**Group B**	**P-value**
	**(control; *n *=24)**	**(anastomosis; *n *=12)**	
Histological findings:			0.836
-Adenocarcinoma	8	3	
- Mucinous cystic neoplasm	2	1	
- Serous cystic neoplasm	0	1	
- Chronic pancreatitis	2	1	
- Pancreatic pseudocyst	2	1	
- Neuroendocrine	10	5	
Pancreatic texture:			0.584
- Soft	18	10	
- Fibrous	6	2	
Main pancreatic duct size:			0.622
- Small	15	8	
- Larger	9	4	
Other surgical procedures:			
- Splenectomy	14	8	0.640
- Nephrectomy	2	0	0.162
- Cholecystectomy^a^	3	1	0.717
- Other procedures^b^	1	0	0.487
Operative time	161 (99–245 min)	192 (155–240 min)	0.024

Quantitative values are given as mean (range).

^a^All these patients had gallstones.

^b^In this case a vascular resection with graft reconstruction was performed.

Food oral intake depended on recovery of gastrointestinal motility and started generally from the third postoperative day. Abdominal drains were removed after a mean duration of 5 days.

PF rate was significantly higher in the group A (control) in which pancreatic leakage occurred in 7 patients (29.1%) compared to group B (anastomosis) where no patient had a PF (p=0.005). They were all pure fistulas, three of grade A and four of grade B, while no grade C fistula occurred. No correlation between PF development and histological findings of the specimens was found. All patients with a grade A fistula were treated conservatively by removing gradually the drain. Patients who had a grade B fistula received total parenteral nutrition (TPN), continuous intravenous somatostatin (6 mg, daily) and antibiotics. In two patients an amylase-rich intra-abdominal collection occurred and was drained by a percutaneous drainage with no severe clinical impact in both cases: this was the reason for classifying them as Grade B (rather than grade C) PF according to recent revisions of the ISGPF classification [17]. These patients were discharged with drains in situ and observed in the outpatient setting. No patient was readmitted.

Other post-operative outcomes were not significant between the two groups as shown in Table 3.

**Table 3:** Post-operative outcomes

**Data**	**Group A** **(control; *n *=24)**	**Group B** **(anastomosis; *n *=12)**	**P-value**
Surgical morbidity:			
- Pancreatic fistula^a^	7 (29.1%)	0	0.002
Grade A	3		
Grade B	4		
Grade C	0		
- Hemorrhage^b^	1 (4.1%)	1 (8.3%)	0.162
Grade A	0	0	
Grade B	0	1	
Grade C	1	0	
- Intra-abdominal abscess	2	0	0.162
- Wound infection	1	0	0.424
Medical morbidity^c^:			
- Cardiac	4	1	0.509
- Pulmonary	1	0	0.487
- Renal	0	1	0.487
- Other	1	1	0.619
Length of hospital stay	9.5 (6–14 days)	8.1 (6–12 days)	0.077
Mortality	0	0	

Quantitative values are given as mean (range).

^a^According to the ISGPF classification [15].

^b^According to the ISGPS classification [16].

^c^Defined as non-surgical post-operative complications.

Postoperative hemorrhage (PH) occurred in two patients of the control group. One patient with normal amylase values from the drain, had a grade C PH due to the rupture of a pseudoaneurysm of the splenic artery after a spleen-preserving DP, which required an angiographic embolization. In the other case the origin of the bleeding was from the retroperitoneal tissue in the spleen site after a DP associated to splenectomy: it was a grade B PH which was treated conservatively by fluids and blood transfusions.

The mean length of hospital stay was higher in group A compared to group B (9.5 vs 8.1 days, respectively), but it was not significant (p=0.077).

Mortality rate was zero in both groups.

## Discussion

DP is a surgical procedure performed with relatively low morbidity and mortality rates in high-volume centers. Surgical outcomes and long-term results have improved widely during the last two decades [18]. However, PF is still the most frequent complication after DP, with an incidence of 5-30% according to the literature [1,3-7], originating from the cut margin of the pancreatic remnant and contributing significantly to morbidity, length of hospital stay and overall costs.

Several approaches to pancreatic stump closure have been described in literature, but none has proved to be the most effective to prevent PF. In a large series by Ferrone et al. [6] different closure techniques were compared as hand-sewn closure, stapler with or without staple line reinforcement, use of free falciform patches and pancreatic duct ligation, but no significant difference in PF rate was found between groups. These data have been confirmed recently by the European multicentric DISPACT trial [7] in which two groups of patients were randomly assigned to stapler or hand-sewn closure of pancreatic remnant with no difference found in PF incidence. The use of artificial patches on the cut margin or the injection of fibrin-glue sealant into the pancreatic duct have also been described [8,9] with good results but larger series are required to demonstrate their efficacy. Recently a new method of stump closure by radiofrequency dissector has been reported with low PF rate, but further prospective studies are needed [10].

As shown in Table 4, few authors described retrospectively their experience of draining the pancreatic stump into a jejunal loop and small series are reported [5,11,12]. More recently, Wagner et al. [14], demonstrated the efficacy of this method comparing the hand-sewn closure to the Roux-en-Y end-to-side pancreato-jejunostomy: they found a zero PF rate in the anastomosis group compared to 20% of PF incidence without anastomosis. However, in that study PF was neither defined nor classified into three grades of severity according to the ISGPF classification [15]. We found same significant results in our series, but with two significant differences: we performed a different type of anastomosis (end-to-end pancreato-jejunostomy) and defined strictly the PF in accordance to the ISGPF classification. The rationale of these encouraging results is based on the assumption that after pancreatic resection the main pancreatic duct is usually visualized and ligated while secondary branches remain always patent owing to their small dimension and this may be a source of pancreatic leakage. This is confirmed by most Authors who reported unchanged PF rates despite they routinely ligated the main pancreatic duct [4,6]. For this reason we minimized the pancreatic secretion by closing the main pancreatic duct while secondary branches were drained by dunking the stump into a jejunal loop: in our opinion the end-to-end anastomosis was the ideal method to achieve a complete drainage of pancreatic juice as the stump is entirely enveloped into the jejunum with this technique compared to the end-to-side anastomosis performed by Wagner et al. [14]. This may explain the low PF rate in our patients. On the other hand, it has to be considered that a pancreatic leakage following small bowel anastomosis could result in a clinically relevant PF (grade B/C PF) and in potentially more hazardous complications (e.g. activation of pancreatic enzymes, bacterial contamination) than leakage after hand-sewn closure of the pancreatic stump. In our series no patient experienced any complication related to the anastomosis, but the statistical power of this study is surely limited by the small sample size of patients, especially for the anastomosis group. For these reasons, at the end of this study, in our institution it currently depends on the surgeon preference to perform the pancreato-jejunostomy especially if it can be carried out safely in selected patients considering the higher operation time and the potential risks of this technique, as described above, despite of its promising results. Moreover there is a clear tendency to perform this operation with a laparoscopic approach, thus some surgeons would prefer anyhow the simple closure by laparoscopic stapler transection rather than open DP with pancreato-jejunostomy.

**Table 4:** Case series reporting pancreato-jejunostomy and other stump closure techniques after distal pancreatectomy

**Authors**	**Study design**	**Variable**	**Sample size n (%)**	**PF rate (%)**	**P-value**
Lillemoe et al. [1]	Retrospective	Pancreato-jejunostomy	10 (4%)	NA^a^	NA
		Hand-sewn closure	204 (87%)	NA	
		Stapled	11 (5%)	NA	
		Both	10 (4%)	NA	
Kleeff et al. [11]	Retrospective	Pancreatico-jejunostomy	24 (8%)	0	0.03
		Hand-sewn closure	97 (32.1%)	9.3	
		Stapled	145 (48%)	15.9	
		Serosal patches	36 (11.9%)	8.3	
Adam et al. [12]	Retrospective	Pancreatico-jejunostomy	27 (65.8%)	7	NS
		Hand-sewn closure	14 (34.2%)	29	
Wagner et al. [14]	Retrospective	Pancreato-jejunostomy	23 (53.5%)	0	0.04
		Hand-sewn closure	20 (46.5%)	20	

*NA* not available, *NS* not significant, *PF* pancreatic fistula.

^a^In this large series the authors reported a total PF rate of 5% with no comparison between methods of stump closure.

Besides different techniques of stump closure, other factors have been considered in PF development after DP: the texture of the pancreatic gland, the use of somatostatin and its analogues (octreotide) and the association with splenectomy. Some studies reported that a non-fibrotic (or soft) pancreas with a small MPD is related to an higher PF rate [19,20]: in our study most cases of both groups had a non-fibrotic pancreas and a small MPD but no PF occurred in the anastomosis groups. The role of somatostatin and its analogues in reducing PF rates after pancreatic surgery is still debated and its use remains controversial [20-24]. Despite a recent Cochrane meta-analysis [22] concluded that the prophylactic use of somatostatin cannot be recommended, other surveys demonstrated its efficacy as consequence of pancreatic exocrine function inhibition after pancreatic surgery [23,24]. On the basis of these studies and in accordance to a more recent meta-analysis [25], we decided to administrate somatostatin analogues prophylactically to all patients, even if most studies cited about its efficacy involved patients undergoing proximal pancreatic resections. The impact of splenectomy on PF development after DP is still controversial [6,26] and we did not find any difference between patients undergoing DP with or without splenectomy.

The operative time was significantly different between the two groups with a mean difference of 31 min, but it was not related to PF development or other post-operative complications.

The length of hospital stay depended primarily on the presence of non-surgical complications.

## Conclusion

In this study we observed the efficacy of the pancreato-jejunostomy to prevent PF after DP compared to the hand-sewn closure of the pancreatic remnant. Despite a higher operation time, it is a safe operation with low morbidity and no mortality rate. However, these are results of a retrospective non-randomized analysis of a small group of patients: larger series are required to confirm these data and several centers must be involved in prospective studies.

## Competing interests

The authors declare that they have no competing interests.

## Authors’ contributions

RLM collected and analyzed clinical data, reviewed the literature and drafted the manuscript; RC, DB participated to the acquisition of data; MS, PC reviewed the manuscript for intellectual content. All authors read and approved the final manuscript.

## Pre-publication history

The pre-publication history for this paper can be accessed here:

http://www.biomedcentral.com/1471-2482/13/23/prepub
